# Serum Concentrations of Transforming Growth Factor-Beta 1 in Predicting the Occurrence of Diabetic Retinopathy in Juvenile Patients with Type 1 Diabetes Mellitus

**DOI:** 10.1155/2013/614908

**Published:** 2013-03-31

**Authors:** Katarzyna Zorena, Ewa Malinowska, Dorota Raczyńska, Małgorzata Myśliwiec, Krystyna Raczyńska

**Affiliations:** ^1^Department of Clinical and Experimental Endocrinology, Institute of Maritime and Tropical Medicine, Medical University of Gdańsk, Powstania Styczniowego 9b, 81-519 Gdynia, Poland; ^2^Department of Immunology, Medical University of Gdańsk, Gdańsk, Poland; ^3^Department and Clinic of Ophthalmology, Medical University of Gdańsk, Gdańsk, Poland; ^4^Department and Clinic of Pediatrics, Diabetology and Endocrinology, Medical University of Gdańsk, Gdańsk, Poland

## Abstract

In the present study, we have decided to evaluate if serum transforming growth factor-beta 1 (TGF-**β**1) concentrations may have diagnostic value in predicting the occurrence of diabetic retinopathy (DR) in juvenile patients with type 1 diabetes mellitus (T1DM). The study included 81 children and adolescents with T1DM and 19 control subjects. All study participants had biochemical parameters examined, underwent an eye examination, and 24-hour blood pressure monitoring. Moreover, serum concentrations of TGF-**β**1 were measured. The group of patients with T1DM and nonproliferative diabetic retinopathy (NPDR) had statistically significant higher serum levels of TGF-**β**1 (*P* = 0.001) as compared to T1DM patients without retinopathy as well as the healthy control subject. The threshold serum TGF-**β**1 concentrations which had a discriminative ability to predict the presence of DR were calculated using the receiver operating characteristic (ROC) curves analysis and amounted to 443 pg/ml. The area under the ROC curve (AUC_ROC_) was 0.80, and its population value was in the range of 0.66 to 0.94. The sensitivity and specificity were calculated to be 72% and 88%, respectively. Our results suggest that TGF-**β**1 serum concentrations may be an additional parameter in predicting the occurrence of DR in juvenile patients with T1DM.

## 1. Introduction

Diabetic retinopathy (DR) is the most common cause of vision loss, and a large number of diabetic patients experience significant vision impairment [1–3]. Usually, in patients with type 1 diabetes mellitus (T1DM) clinically overt changes in the eye fundus do not occur earlier than 5 years of the disease duration. However, after 10 years of the disease, diabetic retinopathy symptoms affect 50% of patients, and after 20–30 years as many as 90% of T1DM patients [2, 3]. Current methods used in medical practice to identify the risk of DR development in children and adolescents with T1DM include the examination of the eye fundus, vision acuity, and colour amblyopia [4, 5]. Many authors emphasise that changes in the eye fundus caused by T1DM are also accompanied by the development of albuminuria which in turn is a marker of diabetic nephropathy [1–3, 6]. However, these clinical markers of vascular damage point to the presence of certain tissue damage, which already gives clinical symptoms such as albuminuria or disturbances of vision. Also, it is common knowledge among ophthalmologists that individual stages of diabetic microangiopathy change smoothly into more advanced. Additionally, when no changes of the retina are seen during ophthalmoscopy, it does not rule out the presence of early histopathological changes in the vessel wall or the presence of abnormal vessel hemodynamics. Apart from the dilatation of retinal blood vessels, the reduction of capillary basal membrane thickness, blood-retina barrier disruption, and a selective loss of pericytes in T1DM patients may also occur [1, 6, 7]. Therefore, there is a strong need for the development of new diagnostic tools which would enable early diagnosis of diabetic complications in juvenile patients with T1DM.

Despite extensive research, the exact pathogenesis of diabetic retinopathy is still unknown. One of its mechanisms is a direct consequence of tissue hypoxia caused by hyperglycemia and the imbalance of local angiogenic factors expression [8–10]. For example, data from our previous studies underline the role of the TNF-*α* and IL-12 overexpression in the pathogenesis of DR in children and adolescents with T1DM [10]. Recently, the role of several growth factors, including transforming growth factor-beta (TGF-*β*), has been emphasised in the pathogenesis of DR [11–13]. TGF-*β* is one of the factors involved in the cellular growth, differentiation and migration, the formation and degradation of extracellular matrix components, chemotactic processes, and apoptosis. In addition, it displays potent strong immunomodulatory activity [8, 12]. It comes in five isoforms, three of which, namely, TGF-*β*1, TGF-*β*2, and TGF-*β*3 are encoded by different genes. The best known among them is TGF-*β*1, which is produced by dendritic cells, leucocytes, and natural killer (NK) cells [14]. Many studies have shown that TGF-*β*1 plays an important role in the pathogenesis of breast cancer, myocardial infarction, autoimmune diseases, osteoporosis, DR, and nephropathy in adults [15–17]. In recent years, however, several studies (including ours) point to the role of TGF-*β*1 in the pathogenesis of microvascular complications in children and adolescents with T1DM [18, 19]. Therefore, in the present study, we have decided to evaluate if serum TGF-*β*1 concentrations may have an additional diagnostic role in predicting the occurrence of DR in juvenile patients with T1DM. 

## 2. Subjects and Methods

### 2.1. Studied Subjects

Eighty-one adolescent patients (44 boys and 37 girls, age range 7–20 yrs) with T1DM from the Department and Clinic of Pediatrics, Diabetology, and Endocrinology at the Medical University of Gdańsk were enrolled into this study. All the patients were under intensive insulin therapy (0.83 ± 0.21 IU of insulin per day/kg of body weight). Diabetes was diagnosed according to the Polish Diabetes Association guidelines which correspond with the guidelines of the European Diabetes Association [20, 21]. Blood pressure was measured using a 24 h blood pressure monitoring (ABPM) method. Various sizes of the cuff were used according to age, weight, and arm circumference of the studied subjects. All the ABPM reports which had less than 80% of technically correct measurements were excluded from the study. Arterial hypertension was diagnosed when mean ABPM values were above the 95th centile for the corresponding age, gender, and height on at least three separate measurements [22]. Ophthalmologic examination was also performed. This included visual acuity tests, intraocular pressure, and anterior segment estimation using the slit lamp (Topcon SL-82, Japan). After local administration of tropicamide (1% solution), the eye fundus was examined using the +90 D lens (Ocular Instruments, USA). A digital camera (Topcon Imaginet 2000, Japan) was used for the fluorescein angiography. The stage of retinopathy was diagnosed according to the guidelines of the International Diabetic Retinopathy Division [4, 5]. Twenty-four hour urine collection was performed three times during the period of 6 months for the evaluation of the daily albumin excretion. The urinary albumin was measured with immunoturbidometric assay using a Tina-quant kit (Boehringer Mannheim GmbH, Germany). Albuminuria was diagnosed when at least two out of three urine samples displayed daily albumin excretion of between 30–299 mg/24 h, collected within 6 months from patients with well-controlled diabetes with no clinical or laboratory signs of ketoacidosis [5, 23]. Serum creatinine was measured using the CREA assay system (Boehringer Mannheim GmbH, Germany). Glycated haemoglobin (HbA1c) was measured with an immunoturbidometric method using a Unimate 3 set (Hoffmann-La Roche AG, Basel, Switzerland). The levels of total cholesterol, HDL cholesterol, LDL cholesterol, and triglycerides were measured using Cormay enzymatic kits (Cormay, Lublin, Poland). Serum C-reactive protein (CRP) concentrations were determined using a highly sensitive testing method (Hs-CRP, Dade Behring, USA). The control group consisted of age and BMI matched 19 healthy children and adolescents (11 boys and 8 girls, age range 6–18 yrs). Written informed consent was obtained from all the participants in the study or from their parents or guardians. This study was approved by The Ethics Committee of The Medical University of Gdańsk (NKEBN/204/2009), and the investigation was carried out in accordance with the principles of the Declaration of Helsinki as revised in 1996.

### 2.2. Serum TGF-*β*1 Measurements

The serum concentrations of TGF-*β*1 were measured using the cytometric bead array (CBA) as instructed by the manufacturer's manual (Plex Flex Single Set, Becton Dickinson, USA). The samples were read in an LSR II flow cytometer using FACS Diva software (Becton Dickinson, USA). Prior to reading, the cytometer was calibrated using the calibration beads included in the test pack (Cytometer Setup Beads). Based on the FSS/SS images, the beads were gated, and fluorescence was read. The analysis was performed using an FCAP Array software (Becton Dickinson, USA).

### 2.3. Statistical Analyses

All statistical calculations were performed using a statistical computer program STATISTICA version 9.0 (http://www.statsoft.com). Quantitative data are presented as an arithmetic mean and standard deviation (SD). To verify whether a quantitative variable was drawn from the population of normal distribution, the Shapiro-Wilk test was performed. The significance of the differences between the control subjects and the diabetic patients was tested with the student *t*-test or *U* Mann-Whitney test when the variables were not normally distributed. Due to multiple comparisons between the groups, Bonferroni correction was applied in calculating the *P* value. To estimate the accuracy of the test, that is, the ability of the test to measure a characteristic in accordance with its actual status, two measures of quantifying the test accuracy were used: sensitivity and specificity. To determine the discriminating threshold value, when results of the test were measured on a continuous scale, receiver operating curve (ROC) was calculated, with the axis of coordinates representing sensitivity and the axis of abscissa representing specificity [24]. The maximum area under the ROC curve (AUC_ROC_) (with values between 0 and 1) was a measure of discriminative power of the test, and the results were reported as means and 95% confidence interval (95% CI) [25]. The *P* value of less than 0.05 was considered statistically significant.

## 3. Results

### 3.1. Clinical Characteristics of All the Study Participants

The clinical characteristics of the studied children and adolescents with T1DM as well as the control subjects are presented in Table 1. The study included 31 T1DM patients with NPDR, aged 15.3 ± 5.4 years and the mean duration of the disease of 9.5 ± 4.9 years, and 57 T1DM patients without retinopathy, aged 14.6 ± 3.9 years and the mean duration of the disease of 6.3 ± 3.5 years. In the NPDR group, 29 patients presented albuminuria, and 11 patients had arterial hypertension. The control group consisted of 19 age-matched (14.2 ± 3.5 years) healthy children. The patients with T1DM and confirmed NPDR had a longer duration of diabetes, higher serum HbA1c levels, higher CRP, higher urinary albumin excretion, and systolic blood pressure values compared to the T1DM patients without retinopathy (*P* < 0.05). No statistically significant differences between the T1DM patients with or without retinopathy were seen in age, serum creatinine levels and the diastolic blood pressure values (*P* > 0.05). However, the patients with T1DM and without retinopathy had significantly higher serum levels of HbA1c and CRP, higher urinary albumin excretion, and higher systolic blood pressure values in comparison with the control group (*P* < 0.05). There was no statistically significant difference in age, serum creatinine levels, and diastolic blood pressure values between the patients with T1DM without retinopathy compared to the control group (*P* > 0.05) (Table 1).

### 3.2. Serum Levels of TGF-*β*1

The patients with T1DM and NPDR displayed statistically significant higher serum levels of TGF-*β*1 (1530 ± 465 pg/mL versus 758 ± 424 pg/mL, *P* = 0.003) as compared to the patients with T1DM but without DR. However, the patients with T1DM but without DR had significantly higher serum levels of TGF-*β*1 compared to the healthy controls subjects (758 ± 424 pg/mL versus 156 ± 49 pg/mL, *P* = 0.001) (Table 2).

### 3.3. Threshold Values of the Studied Parameters Having a Discriminative Ability to Predict the Occurrence of DR in Juvenile Patients with T1DM

In order to determine the threshold values which could have a discriminative ability to predict the occurrence of DR in our T1DM patients, we conducted an ROC analysis. Then, the area under the curve (AUC_ROC_) was calculated for the parameters such as age, duration of diabetes, systolic and diastolic blood pressure, albuminuria, serum HbA1c, CRP, creatinine, and serum TGF-*β*1 levels. The results are presented in (Table 3).

Among all these variables, TGF-*β*1 turned out to have to most discriminative power in predicting the occurrence of DR in the group of juvenile patient with T1DM. The cut-off threshold value was calculated to be 443 pg/mL with AUC_ROC_ of 0.80 (95% CI 0.66–0.94). 

**Figure 1:**
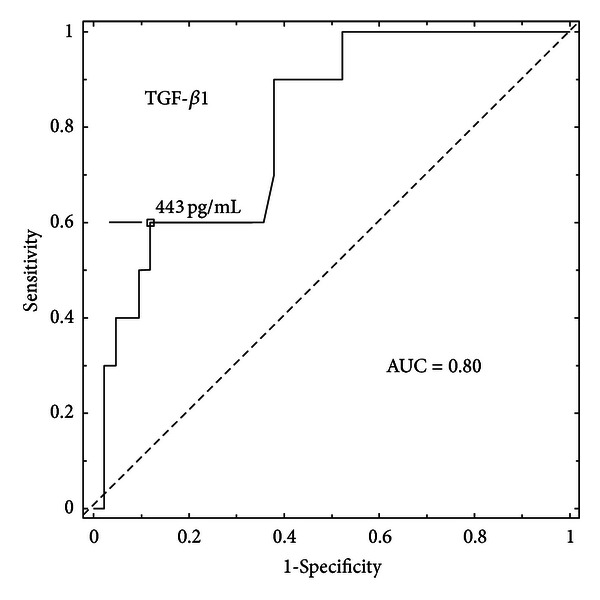
AUC_ROC_ for serum TGF-*β*1.

Sensitivity and specificity were 72 % and 88 %, respectively (Figure 1).

## 4. Discussion

Data from recent studies have shown that TGF-*β*1 is a TGF isoform, which exhibits the strongest relationship with tissue fibrosis [12, 13, 17]. It has also been shown that in long-standing diabetes, TGF-*β*1 overexpression can be the culprit of organ fibrosis, that is, kidney interstitial tissue in the course of diabetic nephropathy [17]. In the retina, TGF-*β*1 is produced by retinal pigment epithelial cells and pericytes, and it is known to be a key profibrotic factor [12]. In proliferative diabetic retinopathy (PDR) and proliferative vitreoretinopathy (PVR), TGF-*β* has been shown to be overexpressed in the vitreoretinal interface [13].

Nevertheless, data on the role of this cytokine in the pathogenesis of vascular diabetic complications in children with T1DM are still scarce [18, 19]. Korpinen et al. (2000) found higher urinary TGF-*β*1 concentrations in children with T1DM compared to control subjects [18]. Also, data from our recent studies have shown increased serum levels of TGF-*β*1 in children with nephropathy. What is more, there was a correlation between serum levels of this cytokine and the advanced glycation end products (AGEs) concentrations in the children and adolescents with T1DM [19]. However, we have not come across any reports concerning serum TGF-*β*1 levels in juvenile patients with diabetic retinopathy. Our hypothesis is that serum TGF-*β*1 concentrations in patients with T1DM may point to the occurrence of retinopathy. Therefore, the aim of our study was to evaluate serum concentrations of this cytokine as well as to determine its threshold values having a discriminative ability in predicting the occurrence of DR in juvenile patients with T1DM. The results of our study have supported our hypothesis and according to our calculations serum TGF-*β*1 concentrations over 443 pg/mL may point to the presence of DR in juvenile patients with T1DM. We have also found that juvenile diabetic patients with NPDR are characterized by higher serum levels of TGF-*β*1 in comparison to the patients without this complication. Moreover, serum TGF-*β*1 concentrations in the patients with T1DM and NPDR was over 10 times higher compared to the healthy controls and about 4 times higher in the diabetic juvenile patients without DR as compared to the healthy controls. According to our data, we conclude that there might be a relationship between TGF-*β*1 overexpression and the presence of DR in juvenile patients with T1DM. The main source of serum TGF-*β*1 are blood plates. However, Loukovaara et al. (2012) have also shown higher intravitreal TGF-*β*1 concentrations in adult T1DM patients with PDR or PDVR, which in turn may be associated with retinal angiogenesis and tissue fibrosis at the vitreoretinal interface [13]. Data from other studies also point to the role of TGF-*β*1 gene polymorphism in the pathogenesis of DR. Beránek et al. (2002) have confirmed a more frequent occurrence of 915G/C (R25P) polymorphism in patients with DR compared to control subjects [26]. Other researchers, in turn, have shown that the activity of urine TGF-*β*1 in diabetic patients increases along with the damage to glomerular cells and renal tubules caused by the locally activated production of TGF-*β*1 [27–29]. This leads to the increase in the renal synthesis of laminin, fibronectin, and collagen. Collagen is prone to glycation, which in turn increases the number of crosslinks in its structure. This results in an increased rigidity of its fibres, their reduced solubility, and reduced susceptibility to enzymatic digestion [29]. 

In clinical practice, there is a strong need for new markers which point to the risk of development or the presence of a specific pathology. Therefore, in the second part of our study, we calculated the threshold TGF-*β*1 concentrations, which would enable us to predict the presence of early-stage diabetic retinopathy in juvenile patients with T1DM. One of the statistical methods of determining the limit values of the parameters under investigation is the analysis of ROC curves. The ROC curve analysis helps to visualize and choose the optimal threshold values that will guarantee the highest decision accuracy [24, 25]. Out of nine tested parameters (age, duration of diabetes, systolic and diastolic blood pressure, albumin excretion rate, serum HbA1c, CRP, TGF-*β*1, and creatinine), it turned out that TGF-*β*1 in serum had the most discriminative power in predicting the occurrence of DR in juvenile patients with T1DM. The cut-off threshold value was calculated to be 443 pg/mL with AUC_ROC_ of 0.80 (95% CI 0.66–0.94). Sensitivity and specificity were 72 % and 88 %, respectively. 

The threshold serum values of TGF-*β*1 were also calculated in other diseases by other investigators [30, 31]. González-Santiago et al. (2011) calculated the threshold TGF-*β*1 value in patients with lung cancer. The cut-point value with a diagnostic power was calculated to be 30.5 pg/mL with 80.5% sensitivity and 64.5% specificity [31]. Nevertheless, our study is the first one where an attempt has been made to define threshold serum TGF-*β*1 concentrations having a discriminative power in predicting the occurrence of DR in patients with T1DM. 

In summary, our results suggest that serum TGF-*β*1 concentrations may be an additional parameter in predicting the occurrence of NPDR in juvenile patients with T1DM.

## Figures and Tables

**Table 1:** Basic clinical parameters in the juvenile patients with T1DM and the control subjects.

Parameters	Patients with T1DM		Healthy control subjects	Statistical significance
	With non-proliferative retinopathy NPDR (+)	Without retinopathy DR (−)		
Number, *n*	31	57	19	
Age, years	15.3 ± 5.4	14.6 ± 3.9	14.2 ± 3.5	NS NS
Diabetes duration, years	9.5 ± 4.9	6.3 ± 3.5	—	<0.001
Albumin extraction rate, mg/24 h	48.03 ± 20.85	10.35 ± 7.5	2.7 ± 1.0	<0.01^†^ <0.001^††^
HbA1c, %	8.9 ± 1.5	7.8 ± 1.3	4.2 ± 0.3	<0.01^†^ <0.0001^††^
CRP, mg/dL	2.4 ± 1.2	1.7 ± 0.8	0.5 ± 0.2	<0.01^†^ <0.001^††^
Creatinine, (*µ*mol/mL)	0.67 ± 0.19	0.66 ± 0.12	0.61 ± 0.11	NS NS
Systolic blood pressure, mm Hg	128.0 ± 12.0	114.0 ± 10.0	100 ± 9.0	<0.001^†^ <0.001^††^
Diastolic blood pressure, mm Hg	72.0 ± 10.0	70.0 ± 8.0	69.0 ± 7.0	NS NS

Data are presented as ± SD statistically significant (*P* < 0.05).

^†^Differences between juvenile patients with and without retinopathy.

^††^Differences between juvenile patients without retinopathy and healthy control subjects.

**Table 2:** Serum TGF-*β*1 levels in T1DM patients and healthy controls.

	Patients with T1DM and NPDR (+)	Patients with T1DM and without DR (−)	Healthy control subjects	Statistical significance
TGF-*β*1 (pg/mL)	1530 ± 465	758 ± 424	156 ± 49	*P* = 0.003^†^ *P* = 0.001^††^

Data are presented as means ± SD.

^†^Differences between juvenile patients with and without retinopathy.

^††^Differences between juvenile patients without retinopathy and healthy control subjects.

**Table 3:** AUC_ROC_ analysis of the studied parameters in juvenile patients with T1DM.

	AUC (95%CI)	Sensitivity	Specificity
Age (years)	0.69 (0.57–0.81)	25.00%	93.80%
Duration of diabetes (years)	0.79 (0.69–0.88)	56.50%	82.50%
Albumin excretion rate (mg/24 h)	0.65 (0.50–0.81)	33.30%	93.80%
HbA1c (%)	0.76 (0.65–0.87)	30.80%	100.00%
SBP (mm/Hg)	0.55 (0.41–0.69)	26.50%	96.90%
DBP (mm/Hg)	0.54 (0.39–0.67)	22.70%	98.80%
Creatinine in serum (*µ*mol/L)	0.53 (0.37–0.68)	19.20%	93.40%
C-reactive protein (mg/dL)	0.67 (0.52–0.81)	37.50%	92.20%
TGF-*β*1	0.80 (0.66–0.94)	72.00%	88.00%
